# Impact of maternal gestational weight gain in twin pregnancies on early childhood obesity risk: A longitudinal birth cohort study

**DOI:** 10.3389/fped.2022.906086

**Published:** 2022-08-02

**Authors:** Sisi Li, Yuhan Qiu, Xi Yuan, Qin Zhang, Mark D. Kilby, Richard Saffery, Philip N. Baker, Li Wen, Chao Tong, Hongbo Qi

**Affiliations:** ^1^State Key Laboratory of Maternal and Fetal Medicine of Chongqing Municipality, The First Affiliated Hospital of Chongqing Medical University, Chongqing, China; ^2^Department of Obstetrics and Gynecology, The Second Affiliated Hospital of Chongqing Medical University, Chongqing, China; ^3^Birmingham Women's and Children's Foundation Trust, Fetal Medicine Centre, Birmingham, United Kingdom; ^4^Institute of Metabolism and Systems Research, College of Medical and Dental Sciences, University of Birmingham, Birmingham, United Kingdom; ^5^Molecular Immunity, Murdoch Children's Research Institute, Royal Children's Hospital, Melbourne, VIC, Australia; ^6^Department of Pediatrics, University of Melbourne, Parkville, VIC, Australia; ^7^College of Life Sciences, University of Leicester, Leicester, United Kingdom; ^8^Chongqing Women and Children's Health Center, Chongqing, China

**Keywords:** childhood obesity, infantile body mass index, gestational weight gain, specific trimester, twin pregnancies

## Abstract

**Objectives:**

To investigate the impact of gestational weight gain (GWG) on the body mass index-for-age z score (BAZ) and obesity risk among twin offspring.

**Methods:**

This study included 263 women who were pregnant with twins and their offspring. Maternal GWG was measured in each trimester, and infant weight and length were measured at 6, 12, and 24 months.

**Results:**

Total GWG was positively correlated with offspring birthweight and BAZ at 6, 12 and 24 months [adjusted β 0.013 (95% CI: 0.008–0.019), 0.028 (95% CI: 0.005–0.050), 0.033 (95% CI: 0.010–0.056) and 0.025 (95% CI: 0.004–0.047), respectively]. Excessive total GWG was related to an increased relative risk (RR) of large for gestational age (LGA) and overweight at 6 and 12 months. Only the second trimester gestational weight gain rate (GWGR) was positively correlated with birthweight (adjusted β 0.380, 95% CI: 0.256–0.504), and RRs of 6.818 (95% CI: 1.568–29.642) and 2.852 (95% CI: 1.466–5.548) were found for LGA and overweight at 12 months, respectively.

**Conclusions:**

Total GWG and the second trimester GWGR were correlated with BAZ and overweight/obesity risk in twin offspring; the impact was obvious in the first year of life and gradually disappeared over time.

**Clinical trial registration:**

ChiCTR-OOC-16008203, Registered on 1 April 2016 at the Chinese Clinical Trial Registry.

## Introduction

Overweight and obesity in children have become increasingly common in recent years (1) and are serious public health issues. In the United States, 23.6% of children and teenagers aged 2–19 years were obese in 2013–2016 (2). According to the latest survey, the prevalence of overweight or obesity was 10.4% among children under 6 years old and 19% among children and adolescents aged 6–17 years in China in 2015–2019 (3). In 2019, ~38 million (6%) of children under 5 years old were overweight or obese globally (4). Childhood obesity is linked to a high risk of a number of health problems later in life, such as predispositions to diabetes mellitus (5), hypertension (6), dyslipidemia (7), and other metabolic diseases (8).

According to the “Developmental Origins of Health and Diseases” theory, intrauterine environmental factors and early life nutrition conditions impact the long-term health of offspring (9). As reported previously, maternal gestational weight gain (GWG), maternal prepregnancy body mass index (pBMI) (10), smoking during pregnancy (11), gestational diabetes mellitus (GDM) (12), milk feeding patterns (13) and adverse childhood experiences (14) have an impact on childhood obesity. Among these factors, GWG, which is a key component of health care during pregnancy, is a significant indicator of the nutritional status in pregnant women. Previous studies have shown that both excessive and inadequate GWG are related to adverse maternal and neonatal outcomes and that excessive GWG is related to childhood overweight in singleton pregnancies (15–19). However, there have been few studies on the correlations between GWG in twin-pregnant women and childhood obesity in twin offspring.

Twin pregnancies have become more prevalent in many countries, partly owing to the increasing maternal age at conception and advances in assisted reproductive technology (ART) (20). Twin fetuses share genetic and intrauterine environments, which makes twin pregnancies a desirable model for evaluating the complex interaction between genetics and the environment (21). In addition, twin offspring are likely to have a preterm birth and low birth weight, which are risk factors for postnatal catch-up growth and excessive weight gain. Therefore, further studies on maternal GWG and offspring growth in twin pregnancies are necessary. In the present study, we investigated the correlations of GWG in each trimester with offspring body mass index (BMI) and early-childhood overweight risk in twin pregnancies from birth to 2 years of age using a longitudinal twin birth cohort.

## Materials and methods

### Recruitment of subjects

**Figure 1:**
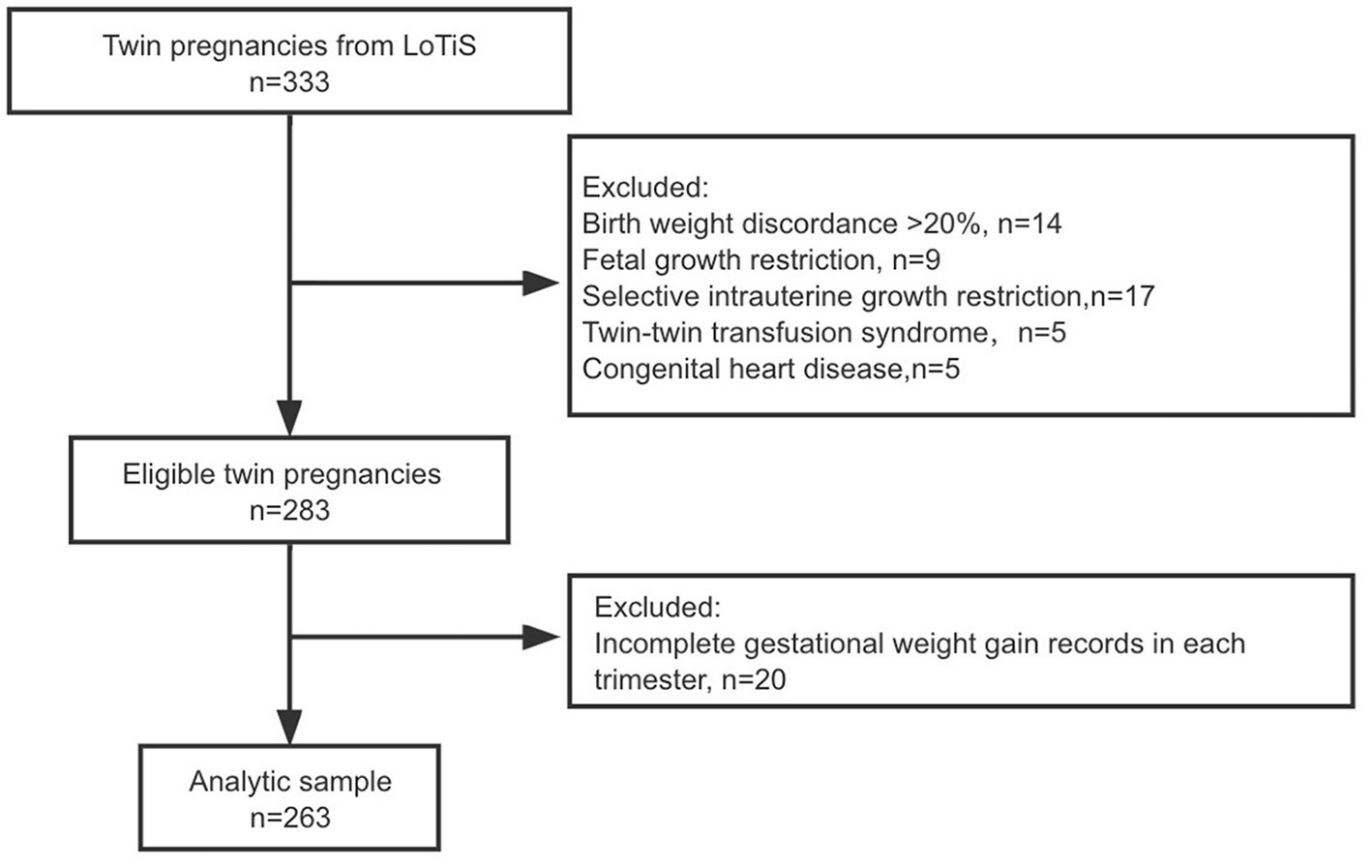
Flowchart showing the selection of twin pairs included in the final analysis of child growth trajectories.

This current study was embedded in the Longitudinal Twin Study (LoTiS), which was established in Chongqing, China, in January 2016 (Trial registration Number: ChiCTR-OOC-16008203) (22). Pregnant women at 11–16 weeks of gestation with twins were recruited for the LoTiS at the First Affiliated Hospital of Chongqing Medical University or the Chongqing Health Center for Women and Children. Subsequent follow-ups were conducted in the second and third trimesters and at delivery, and twin offspring were examined at 6 weeks and at 3, 6, 12, 18, 24, 30, and 36 months of age. The detailed protocol was described previously (23). The Ethics Committee of the First Affiliated Hospital of Chongqing Medical University examined and approved this study (No. 201530). Informed written consent was collected from all participants. A total of 333 LoTiS mother-twin pairs were initially enrolled in the current study. After excluding subjects complicated with severe fetal complications, such as selective intrauterine growth restriction, twin-to-twin transfusion syndrome (*n* = 45), severe congenital malformations of one or both fetuses (*n* = 5), and incomplete GWG records for each trimester (*n* = 20), the final analytic sample included 263 mother-twin pairs. The overall follow-up flow chart is illustrated in Figure 1.

### Data collection

At recruitment, a face-to-face questionnaire interview was used to obtain maternal sociodemographic data, including age, prepregnancy weight, height, educational level, occupation, obstetric history, lifestyle behaviors (smoking, alcohol use), and mode of conception (ART or spontaneous). Maternal pBMI was calculated by prepregnancy weight and height. Chorionicity of a twin pregnancy was identified in the first trimester and confirmed after delivery by placental examination. DNA extracted from umbilical cord blood was used to identify the zygosity of a twin pair by short tandem repeat polymerase chain reaction.

Perinatal outcomes, including pregnancy complications, gestational age at delivery, delivery mode, infant sex, birth weight and length, were derived from medical records. Large for gestational age (LGA) was defined as weight over the 90th percentile for gestational age and sex, according to a Chinese twin population standard (24).

Information about milk feeding patterns in the first 6 months was obtained by interviewing caregivers at the children's follow-up at 6 months.

### Maternal GWG and specific trimester GWGRs

For women who are pregnant with twins, prenatal examination is recommended every 2 or 3 weeks, so we were able to obtain detailed information about GWG. Prepregnancy weight was self-reported by the women at recruitment, and weight was measured by obstetric staff at each prenatal examination until delivery. We collected data on maternal weight at 12 and 28 gestational weeks and prior to delivery from medical records. If a woman had no available weight measurement at 12 or 28 gestational weeks, we adopted linear interpolation between two neighboring weight timepoints to assess the weight for that week. The maternal weight prior to delivery minus the prepregnancy weight was used to calculate total gestational weight gain (TGWG). Specific trimester GWG was obtained as follows: for the first trimester, weight at 12 gestational weeks minus prepregnancy weight; for the second trimester, weight at 28 gestational weeks minus weight at 12 gestational weeks; and for the third trimester, weight before delivery minus weight at 28 gestational weeks. The specific trimester gestational weight gain rate (GWGR) (kilograms per week) was calculated as the specific trimester GWG divided by the corresponding gestational week duration.

The GWG guideline for twin pregnancies implemented in this study was that of the US Institute of Medicine guidelines revised in 2009 (25), whereas there was no definition of specific trimester GWG. Maternal GWG was discovered to be minimal in the first trimester; thus, we classified a GWGR of <0, 0–0.083, and >0.083 kg/wk in the first trimester of pregnancy as inadequate, adequate and excessive, respectively, based on the GWGR distribution in our study population without considering the pBMI. The 75th percentile of the GWGR in the first trimester of pregnancy was 0.083 kg/wk in our study. Moreover, we presumed that the term length for a twin pregnancy was 37 gestational weeks; therefore, for each BMI category, the second- and third-trimester GWGRs were calculated as follows: (IOM recommended total GWG-1)/(37 weeks-12 weeks) (26). Based on China's BMI standard and the IOM guidelines, which have no recommendation for women who are pregnant with twins and were underweight before pregnancy, we classified GWG as inadequate, adequate, and excessive if it was below, within, or above the following recommendations: 16.8–24.5 kg (if pBMI < 24 kg/m^2^), 14.1–22.7 kg (if 24 ≤ pBMI < 28 kg/m^2^) and 11.4–19.1 kg (if pBMI ≥ 28 kg/m^2^). In addition, we classified the GWGR in the second and third trimesters of pregnancy as inadequate, adequate, and excessive if it was below, within, or above the following recommendations: 0.63–0.94 kg/wk (pBMI < 24 kg/m^2^), 0.52–0.87 kg/wk (24 kg/m^2^ ≤ pBMI < 28 kg/m^2^) and 0.42–0.72 kg/wk (pBMI ≥ 28 kg/m^2^).

### Offspring anthropometric assessments

The birth weight and length were measured for all newborns within 1 h of delivery by uniformly trained nurses, and we acquired this information from medical records. At 6 weeks and at 3, 6, and 12 months of corrected age and 18, 24, 30, and 36 months of chronological age, infant weight and length were measured. The corrected age was defined as follows: chronological age (weeks)—(40 weeks- gestational age at birth). Anthropometric data surrounding the indicated time (±7 days) were obtained by professional nurses at the Department of Child Health Care of Chongqing Health Center for Women and Children. The infants were measured with a standing measuring meter or using a digital measuring bed while wearing light clothes and without shoes; the weight and length/height measurements were accurate to within 0.1 kg and 0.1 cm, respectively. Based on the World Health Organization (WHO) Child Growth Standards software (https://www.who.int/childgrowth/software/en/), the BMI of the offspring at each time point was calculated and transformed to z scores for sex and age. A BMI-for-age z score (BAZ) ≥1.035 was used to classify children with overweight/obesity (27). In the current study, anthropometric data at 6, 12, and 24 months were used.

### Statistical analysis

Demographic characteristics and trimester GWGR information were compared according to maternal pBMI status. Continuous data are presented as the means (standard deviations) and were assessed with one-way analysis of variance. Categorical data are presented as frequencies (percentages) and were assessed with the chi-square test or Fisher's exact test. We estimated the linear effect between total GWG or trimester GWGRs and age-specific BAZ by using a generalized estimation equation (GEE) model and the non-linear effect by using a restricted cubic spline (RCS) linear regression model. The GEE model is a regression method that extends the generalized linear model to adjust for the correlation between a set of twins, which is commonly used in twin studies (28–30). The 5, 35, 65, and 95th percentiles of the distribution of specific trimester GWGRs were used in the RCS model. Moreover, we categorized the total GWG and trimester GWGRs into inadequate, adequate, and excessive, and the correlations between the total GWG and specific trimester GWGR categories (with the adequate group as the reference) and the incidence of LGA and childhood overweight were analyzed by using the GEE model. In addition, a subgroup analysis was conducted to observed the modification effect of maternal pBMI on the associations between GWGR and birthweight, age-specific BAZ. The adjusted potential confounders included maternal age, pBMI, education level, chorionicity, mode of conception, parity, smoking before pregnancy, GDM, gestational age at delivery, neonatal sex, birthweight, and milk feeding patterns within 6 months. The effects of all GEE models are presented as beta coefficients or relative risks (RRs), along with their 95% confidence intervals (CIs).

All statistical analyses were conducted in Stata 15.0 (StataCorp, College Station, TX, USA).

## Results

### Basic characteristics

The basic characteristics of the twin-pregnant women and their offspring are shown in Table 1. In our study population, the majority of the participants were mothers who were primiparous, were highly educated, had spontaneous conception, and had a cesarean section (73.8, 72.6, 60.1, and 98.9%, respectively). Over half of the mothers had dichorionic twin pregnancies. At delivery, the average gestational age was 36.49 weeks. Based on their pBMI, 30 (11.4%) women were classified as mothers who were underweight, 176 (66.9%) were classified as mothers who were normal weight, and 57 (21.7%) were classified as mothers who were overweight. The average maternal age tended to be younger in women who were underweight and older in women who were overweight (*P* = 0.001). Although there was no notable difference (*P* = 0.051), the incidence of GDM was lowest in women who were underweight and highest in mothers who were overweight. Other baseline information showed no differences among the three groups. Regarding twin offspring, 56.1% of the newborns were boys, the average birthweight was 2.53 kg, and the average birth length was 46.22 cm. The average birth weight, birth length and incidence of LGA were highest among the neonates born to overweight mothers (all *P* < 0.05). There were no differences between the groups in the BAZ and milk feeding patterns within 6 months. Due to the unavoidable loss to follow-up, the follow-up numbers of infants were 469 at 6 months, 431 at 12 months and 397 at 24 months.

**Table 1:** Basic characteristics of the study population by maternal pre-pregnancy weight status.

		**Maternal pre-pregnancy weight status**			
**Variable**	**Total** ***N*** **= 263**	**Underweight *n* = 30**	**Normal weight *n* = 176**	**Overweight *n* = 57**	***P*** **-value**
**Mothers**	**Mean (SD)**	**Mean (SD)**	**Mean (SD)**	**Mean (SD)**	
Age (y)	29.35 (3.88)	27.37 (3.45)	29.29 (3.80)	30.56 (3.94)	0.001^*^
Prepregnancy BMI (kg/m^2^)	21.82 (3.09)	17.54 (0.75)	21.12 (1.54)	26.24 (2.38)	<0.001^*^
Prepregnancy weight (kg)	55.25 (8.55)	44.92 (3.21)	53.39 (5.10)	66.46 (7.73)	<0.001^*^
Gestational age (wks)	36.49 (1.53)	36.68 (1.63)	36.41 (1.55)	36.63 (1.40)	0.489^*^
	***n*** **(%)**	***n*** **(%)**	***n*** **(%)**	***n*** **(%)**	
**Age (y)**					0.054^#^
<25	27 (10.3)	7 (23.3)	17 (9.7)	3 (5.3)	
25–29	116 (44.1)	15 (50.0)	80 (45.4)	21 (36.8)	
30–34	92 (34.9)	7 (23.3)	61 (34.7)	24 (42.1)	
≥ 35	28 (10.7)	1 (3.3)	18 (10.2)	9 (15.8)	
**Parity**					0.368^#^
Primiparous	194 (73.8)	22 (73.3)	134 (76.1)	38 (66.7)	
Multiparous	69 (26.2)	8 (26.7)	42 (23.9)	19 (33.3)	
**Maternal education level**					0.971^#^
≤ Senior high school	72 (27.4)	8 (26.7)	49 (27.8)	15 (26.3)	
>Senior high school	191 (72.6)	22 (73.3)	127 (72.2)	42 (73.7)	
**Smoked before pregnancy**					1.000^#^
Yes	22 (8.4)	2 (6.7)	15 (8.5)	5 (8.8)	
No	241 (91.6)	28 (93.3)	161 (91.5)	52 (91.2)	
**Mode of conception**					0.288^#^
Spontaneous	158 (60.1)	22 (73.3)	103 (58.5)	33 (57.9)	
ART	105 (39.9)	8 (26.7)	73 (41.5)	24 (42.1)	
**Chorionicity**					0.754^#^
MC	102 (38.8)	11 (36.7)	71 (40.3)	20 (35.1)	
DC	161 (61.2)	19 (63.3)	105 (59.7)	37 (64.9)	
GDM					0.051^#^
Yes	80 (30.4)	4 (13.3)	54 (30.7)	22 (38.6)	
No	183 (69.6)	26 (86.7)	122 (69.3)	35 (61.4)	
**Delivery mode**					0.409^#^
Spontaneous vaginal delivery	3 (1.1)	1 (3.3)	2 (1.1)	0 (0.0)	
Cesarean section	260 (98.9)	29 (96.7)	174 (98.9)	57 (100.0)	
**Infants**	***n*** **= 526**	***n*** **= 60**	***n*** **= 352**	***n*** **= 114**	
	**Mean (SD)**	**Mean (SD)**	**Mean (SD)**	**Mean (SD)**	
Birth weight (kg)	2.53 ± 0.40	2.52 ± 0.39	2.49 ± 0.39	2.64 ± 0.40	0.002^*^
Birth length (cm)	46.22 ± 2.23	46.36 ± 1.97	46.05 ± 2.30	46.68 ± 2.09	0.029^*^
**Childhood BAZ**					
6 months ^a^	0.39 ± 0.94	0.33 ± 0.98	0.37 ± 0.96	0.51 ± 0.83	0.395^*^
12 months ^b^	0.13 ± 0.92	−0.18 ± 0.94	0.13 ± 0.93	0.25 ± 0.88	0.254^*^
24 months ^c^	0.05 ± 0.83	−0.08 ± 0.70	0.04 ± 0.84	0.13 ± 0.85	0.431^*^
	***n*** **(%)**	***n*** **(%)**	***n*** **(%)**	***n*** **(%)**	
**Sex**					0.918^#^
Male	295 (56.1)	34 (56.7)	199(56.5)	62 (54.4)	
Female	231 (43.9)	26(43.3)	153 (43.5)	52 (45.6)	
**LGA at birth**					0.003^#^
Yes	16 (3.0)	2 (3.3)	5 (1.4)	9 (7.9)	
No	510 (97.0)	58 (96.7)	347 (98.6)	105 (92.1)	
**Milk feeding pattern within 6 months**					0.576^#^
Exclusively breastfed	82 (17.5)	7 (12.5)	58 (18.0)	17 (18.9)	
Exclusively formula fed	211 (45.0)	26 (46.4)	150 (46.4)	35 (38.9)	
Mixed feedings	176 (37.5)	23 (41.1)	115 (35.6)	38 (42.2)	

ART, assisted reproductive technology; BAZ, body mass index-for-age z score; DC, dichorionic; BMI, body mass index; GDM, gestational diabetes mellitus; GWG, gestational weight gain; LGA, large for gestational age; MC, monochorionic.

* *P*-value from one-way analysis of variance;

# *P*-value from chi-square test;

a 469 infants were assessed;

b 431 infants were assessed;

c 397 infants were assessed.

We compared the demographic and clinical characteristics between the included participants and initially enrolled participants, and the results showed that the characteristics were not different (Supplementary Table 1).

### GWGR characteristics

The details of the total GWG and the specific trimester GWGR are depicted in Supplementary Table 2. Mothers who were underweight had the highest average total GWG, whereas those who were overweight had the lowest. In the first, second, and third trimesters, the average GWGR was 0.08, 0.69, and 0.70 kg/wk, respectively.

In the first trimester, according to the GWGR distribution, 40.7% of the pregnant women had an inadequate GWGR (≤ 0 kg/wk), 45.3% had an adequate GWGR (0–0.25 kg/wk), and 14.1% had an excessive GWGR (≥0.25 kg/wk). In the second trimester, 41.4, 48.3, and 10.3% of the study group had inadequate, adequate, and excessive GWGRs, respectively. No differences were found between the groups in the distribution of the first two trimester GWGRs. In the third trimester, 35.7, 42.2, and 22.1% of the study group had inadequate, adequate, and excessive GWGRs, respectively. The percentage of inadequate GWGRs was lowest in women who were overweight in the third trimester (*P* = 0.007).

### GWG and offspring birthweight and age-specific BAZ

The univariate and multiple regression analyses of the relationship between total GWG and offspring birthweight and BAZ at 6, 12, and 24 months are summarized in Table 2. In an unadjusted model, total GWG was positively correlated with offspring birthweight and BAZ at 6, 12, and 24 months. After adjusting for maternal age, pBMI, parity, education level, smoking before pregnancy, GDM, gestational age, mode of conception, chorionicity and milk feeding patterns within 6 months, the positive associations were still significant between total GWG and birthweight and BAZ at 6, 12, and 24 months [adjusted β 0.013 (95% CI: 0.008–0.019), 0.028 (95% CI: 0.005–0.050), 0.033 (95% CI: 0.010–0.056) and 0.025 (95% CI: 0.004–0.047), respectively).

**Table 2:** Unadjusted and adjusted correlations between total gestational weight gain and birthweight and age-specific BAZ.

**Outcomes**	**Model 1 β (95% CI)**	**Model 2 β (95% CI)**
Birthweight	0.023 (0.015–0.032)	0.013 (0.008–0.019)^a^
**BAZ**		
6-months	0.022 (0.001–0.044)	0.028 (0.005–0.050)
12-months	0.027 (0.004–0.050)	0.033 (0.010–0.056)
24-months	0.021 (0.001–0.042)	0.025 (0.004–0.047)

BAZ, body mass index z score. Model 1: crude β; Model 2: adjusted β. Confounding factors included maternal age, pBMI, parity, education level, smoking before pregnancy, GDM, gestational age, mode of conception, chorionicity and infant milk feeding patterns within 6 months.

a Confounding factors included maternal age, pBMI, parity, education level, smoking before pregnancy, GDM, gestational age, mode of conception and chorionicity.

### GWG and risk of LGA and early childhood overweight

Before we analyzed the impact of GWG on childhood overweight, we assessed the concordance rate of the growth patterns in twin offspring. The concordance rate of both infants being normal or overweight was higher in monozygotic (MZ) twins than in dizygotic (DZ) twins, and the concordance rate increased with age in both MZ and DZ twins (Supplementary Table 3).

The univariate and multiple logistic regression analyses of the correlation of total GWG categories with the risk of LGA and childhood overweight are shown in Table 3. After adjusting for possible confounders, excessive GWG was correlated with increased RRs of 5.947 [95% confidence interval (CI): 1.820, 25.678], 2.069 (95% CI: 1.290, 3.318) and 3.964 (95% CI: 1.949–15.190) for LGA at birth and overweight at 6 and 12 months, respectively, when compared to the reference group.

**Table 3:** Unadjusted and adjusted correlations between total gestational weight gain and LGA and age-specific overweight.

**Outcomes**	**Study factors GWG**	**Model 1 RR (95% CI)**	**Model 2 RR (95% CI)**
LGA at birth	Inadequate	0.848 (0.206–3.467)	0.581 (0.116–2.921) ^a^
	Excessive	6.417 (1.780–23.133)	5.947 (1.820–25.678) ^a^
**Overweight**			
6-months	Inadequate	1.279 (0.819–1.997)	1.261 (0.791–2.012)
	Excessive	2.217 (1.373–3.581)	2.069 (1.290–3.318)
12-months	Inadequate	1.175 (0.618–2.234)	1.318 (0.700–2.480)
	Excessive	3.200 (1.720–5.950)	3.964 (1.949–15.190)
24-months	Inadequate	0.791 (0.346–1.807)	0.782 (0.358–1.707)
	Excessive	1.968 (0.847–4.572)	1.613 (0.635–4.098)

LGA, large for gestational age; RR,relative risk; Inadequate, inadequate GWG was below the following recommendations: 16.8–24.5 kg (if pBMI < 24 kg/m^2^), 14.1–22.7 kg (if 24 ≤ pBMI < 28 kg/m^2^) and 11.4–19.1 kg (if pBMI ≥ 28 kg/m^2^); Excessive, excessive GWG was above the following recommendations: 16.8–24.5 kg (if pBMI < 24 kg/m^2^), 14.1–22.7 kg (if 24 ≤ pBMI < 28 kg/m^2^) and 11.4–19.1 kg (if pBMI ≥ 28 kg/m^2^). Model 1: crude RR; Model 2: adjusted RR. Confounding factors included maternal age, pBMI, parity, education level, smoking before pregnancy, GDM, gestational age, mode of conception, chorionicity and infant milk feeding patterns within 6 months.

a Confounding factors included maternal age, pBMI, parity, education level, smoking before pregnancy, GDM, gestational age, mode of conception and chorionicity.

### GWGR and offspring birthweight/BAZ

To investigate which GWG period had a key impact on offspring growth, correlations between trimester GWGRs and birthweight and childhood BAZ were determined. The results indicated that only the second trimester GWGR was positively correlated with offspring birthweight (Table 4). The mean birthweight increased by 0.38 kg when the second trimester GWGR increased by 1 kg/wk (95% CI: 0.256–0.504) after controlling for potential confounders. In addition, we detected whether non-linear effects existed between specific trimester GWGRs and offspring birthweight or childhood BAZ. Data pooling showed estimations and 95% CIs for the GWGR in different trimesters with birthweight and BAZ at 6, 12, and 24 months by RCS linear regression models among the total population; potential non-linear correlations of the second trimester GWGR with offspring BAZ at 6 and 12 months; and potential non-linear correlations of the third trimester GWGR with offspring birthweight (Supplementary Figures 1–3).

**Table 4:** Unadjusted and adjusted correlations between trimester GWGRs and birthweight and age-specific BAZ.

**Outcomes**	**Model 1 β (95% CI)**	**Model 2 β (95% CI)**
**First trimester**		
Birthweight	0.040 (−0.156 to 0.236)	0.060 (−0.061 to 0.180) ^a^
**BAZ**		
6-months	0.331 (−0.149 to 0.811)	0.244 (−0.228 to 0.717)
12-months	0.441 (−0.036 to 0.918)	0.288 (−0.168 to 0.745)
24-months	0.457 (−0.015 to 0.929)	0.392 (−0.073 to 0.857)
**Second trimester**		
Birthweight	0.329 (0.123–0.535)	0.380 (0.256–0.504) ^a^
**BAZ**		
6-months	0.165 (−0.351 to 0.682)	0.338 (−0.185 to 0.861)
12-months	0.245 (−0.288 to 0.777)	0.454 (−0.066 to 0.974)
24-months	0.167 (−0.325 to 0.660)	0.350 (−0.149 to 0.848)
**Third trimester**		
Birthweight	0.053 (−0.084 to 0.190)	−0.023 (−0.108 to 0.062) ^a^
**BAZ**		
6-months	0.199 (−0.132 to 0.530)	0.267 (−0.062 to 0.595)
12-months	0.130 (−0.221 to 0.480)	0.198 (−0.136 to 0.532)
24-months	0.081 (−0.246 to 0.408)	0.088 (−0.240 to 0.416)

BAZ, body mass index z score. Model 1: crude β; Model 2: adjusted β. Confounding factors included maternal age, pBMI, parity, education level, smoking before pregnancy, GDM, gestational age, mode of conception, chorionicity and infant milk feeding patterns within 6 months.

a Confounding factors included maternal age, pBMI, parity, education level, smoking before pregnancy, GDM, gestational age, mode of conception and chorionicity.

In the subgroup analyses, this positive correlation remained evident in mothers who were underweight (β 0.723, 95% CI: 0.335–1.111), normal weight (β 0.256, 95% CI: 0.102–0.409) and overweight (β 0.550, 95% CI: 0.334–0.766) (Supplementary Figure 4). In addition, the third trimester GWGR was found to be positively correlated with BAZ at 6 months (β 0.767, 95% CI: 0.154–1.381), 12 months (β 0.946, 95% CI: 0.231–1.660) and 24 months (β 0.893, 95% CI: 0.118–1.668) only among overweight mothers.

### GWGR and risk of LGA and early childhood overweight

The correlations of unadjusted and adjusted RRs for LGA and overweight at 6, 12, and 24 months with excessive or inadequate trimester GWGRs are depicted in Table 5. In the general population, excessive GWGR only in the second trimester was correlated with an increased RR of 6.818 (95% CI: 1.568–29.642) and 2.852 (95% CI: 1.466–5.548) for LGA and overweight at 12 months, respectively, after controlling for confounding factors. There were no differences between inadequate trimester GWGRs and LGA or overweight at 6, 12, and 24 months.

**Table 5:** Unadjusted and adjusted correlations between excessive or inadequate trimester GWGR and LGA and age-specific overweight.

**Outcomes**		**Model 1 RR** **(95% CI)**	**Model 2 RR** **(95% CI)**
**First trimester**			
LGA at birth	Inadequate	0.407 (0.119–1.390)	0.335 (0.083–1.344) ^a^
	Excessive	0.563 (0.147–2.153)	0.507 (0.134–1.922) ^a^
**Overweight**			
6-months	Inadequate	0.728 (0.476–1.114)	0.793 (0.511–1.230)
	Excessive	0.839 (0.514–1.370)	0.862 (0.521–1.429)
12-months	Inadequate	0.668 (0.354–1.259)	0.649 (0.332–1.270)
	Excessive	1.090 (0.576–2.063)	1.016 (0.532–1.939)
24-months	Inadequate	0.872 (0.372–2.046)	0.919 (0.370–2.280)
	Excessive	1.533 (0.654–3.593)	1.460 (0.647–3.293)
**Second trimester**			
LGA at birth	Inadequate	0.504 (0.062–4.089)	0.132 (0.009–1.892) ^a^
	Excessive	2.889 (1.891–9.365)	6.818 (1.568–29.642) ^a^
**Overweight**			
6-months	Inadequate	1.370 (0.830–2.258)	1.245 (0.735–2.109)
	Excessive	1.352 (0.847–2.158)	1.342 (0.836–2.154)
12-months	Inadequate	1.668 (0.762–3.650)	1.355 (0.587–3.129)
	Excessive	2.658 (1.473–4.796)	2.852 (1.466–5.548)
24-months	Inadequate	0.981 (0.340–2.834)	0.863 (0.315–2.364)
	Excessive	1.506 (0.712–3.185)	1.340 (0.617–2.914)
**Third trimester**			
LGA at birth	Inadequate	0.606 (0.110–3.339)	0.442 (0.095–2.049) ^a^
	Excessive	2.090 (0.569–7.675)	1.847 (0.534–6.389) ^a^
**Overweight**			
6-months	Inadequate	0.840 (0.520–1.358)	0.789 (0.498–1.274)
	Excessive	0.767 (0.469–1.253)	0.734 (0.444–1.215)
12-months	Inadequate	0.622 (0.315–1.242)	0.547 (0.269–1.112)
	Excessive	0.752 (0.401–1.407)	0.716 (0.378–1.358)
24-months	Inadequate	0.682 (0.282–1.652)	0.630 (0.250–1.587)
	Excessive	0.949 (0.426–2.112)	0.841 (0.391–1.813)

LGA, large for gestational age; Inadequate, inadequate GWGR was below 0 kg/wk in the first trimester and below the following recommendations in the second and third trimesters: 0.63–0.94 kg/wk (pBMI < 24 kg/m^2^), 0.52–0.87 kg/wk (24 kg/m^2^ ≤ pBMI < 28 kg/m^2^) and 0.42–0.72 kg/wk (pBMI ≥ 28 kg/m^2^); Excessive, excessive GWG was above 0.083 kg/wk in the first trimester and above the following recommendations in the second and third trimesters: 0.63–0.94 kg/wk (pBMI < 24 kg/m^2^), 0.52–0.87 kg/wk (24 kg/m^2^ ≤ pBMI < 28 kg/m^2^) and 0.42–0.72 kg/wk (pBMI ≥ 28 kg/m^2^). Model 1: crude RR; Model 2: adjusted RR. Confounding factors included maternal age, pBMI, parity, education level, smoking before pregnancy, GDM, gestational age, mode of conception, chorionicity and infant milk feeding patterns within 6 months.

a Confounding factors included maternal age, pBMI, parity, education level, smoking before pregnancy, GDM, gestational age, mode of conception and chorionicity.

## Discussion

In this study, we examined the relationship between total GWG and offspring growth in twin pregnancies, and the results demonstrated that increased total GWG was related to higher offspring birthweight and BAZ in the first 2 years. Excessive total GWG was correlated with a higher risk of overweight at 6 and 12 months. Second, we investigated the impact of the GWGR in each trimester on twin offspring growth, and the results illustrated that only the second trimester GWGR was positively associated with offspring birthweight. Moreover, an excessive GWGR only in the second trimester was correlated with a higher risk of overweight at 12 months.

Previous twin studies have reported that genetic factors have a significant influence on BMI at all ages based on a twin model (31), but early life factors may have a stronger effect than genetic factors on childhood obesity (32). The environmental factors shared by cotwins had a noticeable effect in infancy and early childhood, but this effect was less pronounced after mid-childhood and disappeared by adulthood (33), which may mirror the growing independence of children from their parents and family environment, leading to more individualistic exercise and eating behaviors (34). In this study, we found that the concordance rate of the growth pattern in twins was higher with increasing age, suggesting that early life factors played an important role in childhood growth and development.

Recent studies have indicated that maternal GWG is correlated with offspring growth. Xie et al. discovered a significantly positive relationship between GWG and high neonatal birthweight among a Southwest Chinese population (35). A positive relationship between maternal total GWG and childhood BMI was found among different populations at different ages (10, 36). Zhang et al. reported that excessive GWG, only in the first trimester, was related to a higher risk of overweight or obesity in offspring at 12 months (37). Likewise, Diesel et al. found that excessive total GWG was related to more than double the risk of childhood obesity at 3 years old compared with no overall excessive GWG in the U.S. (38). Bodnar et al. found that higher gestational weight gain in twin pregnancies was positively associated with the risk of excess postpartum weight increase and childhood overweight/obesity in a nationally representative U.S. birth cohort (39). Our findings are in accordance with previous studies that suggested an association of higher total GWG with increased offspring BAZ at 6, 12, and 24 months and with increased RR for LGA and overweight at 6 and 12 months. A possible hypothesis is that higher GWG indicates excess nutrients *in utero*, thus exposing the fetus to excessive levels of fatty acids and glucose, which may increase fetal insulin production and consequently result in increased adiposity (40), although it might be beneficial for age-dependent decline brain health (41).

Since maternal weight gain is not uniform throughout gestation, therefore, some researchers have concentrated on specific trimester GWG and offspring growth. Previous studies of the effect of trimester GWG and offspring growth have been inconsistent. In singletons, a study by Xiong et al. suggested that an excessive GWGR in each trimester was positively related to offspring BAZ and the risk of overweight or obesity in early childhood (19). Hivert et al. concluded that a higher GWGR in each trimester of pregnancy was related to higher birthweight, whereas only a higher GWGR in the first two trimesters was related to increased BAZ and risk of obesity in mid-childhood, and the findings were inconsistent among mothers in different BMI categories (17). Pugh et al. found that an excessive GWGR in the second and third trimesters increased the risk of LGA in offspring (42). A previous study in twin pregnancies reported that only the second trimester GWG was related to fetal growth, driven by a correlation with abdominal circumference in the early second trimester and long bones in the late second trimester (43). Our findings suggested that, overall, only the second trimester GWGR was positively related to infant birthweight and was correlated with a higher risk of LGA and overweight at 12 months in twins, which was consistent with our previous research reporting that second trimester GWG was positively related to offspring birthweight among dichorionic twin pregnancies (44). This finding could be comforting to women who have a sufficient prepregnancy nutritional status but restricted weight gain in the first trimester because of increased vomiting and nausea symptoms, which are common in twin pregnancies (45). A biological explanation for this phenomenon is that the second trimester is the most critical stage for organogenesis, including the growth of the heart, liver, muscles and bones (17). The development of fetal adipose tissue also begins around the same time, with the first “fat lobules” appearing between 14 and 24 weeks of gestation, when nutritional, hormonal, and epigenetic signals can be affected by the mother and may lead to permanent alterations in the adipose tissue of offspring (46). Thus, the second trimester GWG may promote the growth of both organs and adipose tissue, supporting our observations related to offspring BAZ and obesity. In the third trimester, a number of complicating factors, such as intrauterine environment or placental insufficiency, may limit fetal growth in twin pregnancies, attenuating the correlation of GWG with fetal growth (47).

Since women who are pregnant with twins and have inadequate GWG are prone to preterm delivery, their offspring are prone to low birthweight and are more likely to undergo catch-up growth after birth, which can also lead to overweight later in life (48). In the present study, the proportion of preterm infants was high, and the birthweight of the infants was low. Given that infants with either low or high birthweight can be overweight in childhood, the long-term impacts of birthweight exhibit a U-shaped curve (49). Thus, we conducted an association analysis between inadequate GWG and childhood overweight and found no associations, indicating that overweight or obesity in preterm infants may be caused by other factors and warrants further study.

The strengths of this study warrant discussion. To our knowledge, this was the first study on the correlations of trimester GWGRs with offspring growth from birth to 2 years of age by using a longitudinal twin pregnancy birth cohort. Additionally, a stratified analysis was used to assess the relationship between GWG/GWGRs and birthweight/BAZ at different pBMI levels, making the study more reliable, accurate and comprehensive. Furthermore, our study converted weight, height and BMI into z scores based on the WHO growth criteria, which is a commonly used reference standard in China.

However, several limitations need to be noted. First, the participants were asked to recall their prepregnancy height and weight, which could lead to recall bias, although previous studies have revealed that the error has a minor effect on the reported associations and conclusions (50, 51). Second, there may be residual confounders, which we did not consider in this analysis. Finally, we only followed up the offspring growth to 2 years of age, and the long-term impacts of GWG are unknown. Therefore, LoTiS will continue to monitor the growth and development of twins and further elucidate the impact of GWG on growth in twins.

## Conclusion

In conclusion, the impact of total GWG on BMI and the risk of obesity for offspring was obvious in the first year of life and gradually disappeared as the twin infants grew older. Additionally, only the second trimester GWGR was correlated with offspring birthweight and the risk of early-childhood obesity in twins. The findings may motivate further study to investigate the guidelines of GWG/GWGRs for twin-pregnant women of diverse ethnicities as well as the development of intervention strategies aimed at controlling the rapidly rising prevalence of childhood obesity.

## Data availability statement

The original contributions presented in the study are included in the article/Supplementary material, further inquiries can be directed to the corresponding author/s.

## Ethics statement

The studies involving human participants were reviewed and approved by Ethics Committee of the First affiliated hospital of Chongqing Medical University. Written informed consent to participate in this study was provided by the participants' legal guardian/next of kin.

## Author contributions

CT and HQ designed and conceived the study. SL, LW, and YQ collected and analyzed the data. RS, MK, and PB interpreted the results. CT, LW, and HQ provided the funding resources. SL and LW wrote the draft. CT, MK, and PB edited the manuscript. All authors contributed to the article and approved the submitted version.

## Funding

This research was supported by the National Natural Science Foundation of China (U21A20346, 81520108013, 82001580, and 82171662), Chongqing Science and Technology Commission (cstc2021ycjh-bgzxm0192), Chongqing Health Committee (2019GDRC012), and Chongqing Education Commission (KJZD-K202100404).

## Conflict of interest

The authors declare that the research was conducted in the absence of any commercial or financial relationships that could be construed as a potential conflict of interest.

## Publisher's note

All claims expressed in this article are solely those of the authors and do not necessarily represent those of their affiliated organizations, or those of the publisher, the editors and the reviewers. Any product that may be evaluated in this article, or claim that may be made by its manufacturer, is not guaranteed or endorsed by the publisher.

## Abbreviations

pBMI: prepregnancy body mass index
GWG: gestational weight gain
GDM: gestational diabetes mellitus
ART: assisted reproductive technology
BMI: body mass index
LoTiS: longitudinal twin cohort study
LGA: large for gestational age
TGWG: total gestational weight gain
GWGR: gestational weight gain rate
WHO: World Health Organization
BAZ: body mass index-for-age z score
GEE: generalized estimation equation
RCS: restricted cubic spline
MZ: monozygotic
DZ: dizygotic
RR: relative risk
CI: confidence interval
DC: dichorionic
MC: monochorionic.
