# Supplementary material for: Impact of maternal gestational weight gain in twin pregnancies on early childhood obesity risk: A longitudinal birth cohort study
Source: Front Pediatr. 2022 Aug 2;10:906086. doi: 10.3389/fped.2022.906086 (PMC9378839; doi:10.3389/fped.2022.906086)
Supplement: Supplementary file 1 [file Table_1.DOCX]

**Supplementary Table 1.** Comparison of included and all population in terms of demographic characteristics.

| Groups | Included | All | P value |
| --- | --- | --- | --- |
| Mothers | n = 263 | n = 333 |  |
| Age (years old) | 29.35± 3.88 | 29.49±4.18 | 0.603 |
| < 25 | 27 (10.3) | 36 (10.8) |  |
| 25-29 | 116 (44.5) | 144 (43.2) |  |
| 30-34 | 92 (35.0) | 117 (35.1) |  |
| ≥ 35 | 28 (10.7) | 36 (10.8) |  |
| Prepregnancy BMI (kg/m2) | 21.82 ± 3.09 | 21.04±3.06 | 0.796 |
| Prepregnancy weight (kg) | 55.25 ± 8.55 | 54.17±8.83 | 0.793 |
| Gestational weight gain (kg) | 18.06 ± 4.97 | 18.01±5.39 | 0.143 |
| Parity |  |  | 0.910 |
| Primiparous | 194 (73.8) | 247 (74.2) |  |
| Multiparous | 69 (26.2) | 86 (25.8) |  |
| Maternal education level |  |  | 0.795 |
| ≤ Senior high school | 72 (27.4) | 88 (26.4) |  |
| > Senior high school | 191 (72.6) | 245 (73.6) |  |
| Smoking use before pregnancy |  |  | 0.882 |
| Yes | 22 (8.4) | 29 (8.7) |  |
| No | 241 (91.6) | 304 (91.3) |  |
| Mode of conception |  |  | 0.769 |
| Spontaneous | 158 (60.1) | 204 (61.3) |  |
| ART | 105 (39.9) | 129 (38.7) |  |
| Chorionicity |  |  | 0.341 |
| MC | 102 (38.8) | 142 (42.6) |  |
| DC | 161 (61.2) | 191 (57.4) |  |
| GDM |  |  | 0.856 |
| Yes | 80 (30.4) | 99 (29,7) |  |
| No | 183 (69.6) | 234 (70.3) |  |
| Gestational age (wk) | 36.49 ± 1.53 | 36.35 ± 1.59 | 0.278 |
| Infants | n = 526 | n = 666 |  |
| Sex |  |  | 0.896 |
| Male | 295 (56.1) | 371 (55.7) |  |
| Female | 231 (43.9) | 295 (44.3) |  |
| Birth weight (kg) | 2.53 ± 0.40 | 2.46 ± 0.44 | 0.145 |
| Birth length (cm) | 46.22 ± 2.23 | 45.91 ± 2.48 | 0.178 |
| LGA at birth |  |  | 0.848 |
| Yes | 16 (3.0) | 19 (2.9) |  |
| No | 510 (97.0) | 647 (97.1) |  |

ART, assisted reproductive technology; BMI, body mass index; BAZ, body mass index z scores; DC, dichorionic; GDM, gestational diabetes mellitus; LGA, large for gestational age; MC, monochorionic.

**Supplementary Table 2.** Total GWG and trimester-specific GWGR and distribution of inadequate, adequate, and excessive GWGR according to maternal prepregnancy BMI.

| Variable | Total  (n = 263) | Underweight  (n = 30) | Normal weight  (n = 176) | Overweight  (n = 57) | P-value |
| --- | --- | --- | --- | --- | --- |
| Total GWG (kg) | 18.06 ± 4.97 | 20.45 ± 6.09 | 18.08 ± 4.52 | 16.72 ± 5.27 | 0.004 |
| First trimester (kg/wk) | 0.08 ± 0.23 | 0.11 ± 0.17 | 0.08 ± 0.24 | 0.05 ± 0.20 | 0.463 |
| Inadequate (<0) | 107 (40.7) | 10 (33.3) | 73 (41.5) | 24 (42.1) | 0.350 |
| Adequate (0-75th percentile) | 119 (45.3) | 16 (53.3) | 74 (42.1) | 29 (50.9) |  |
| Excessive (>75th percentile) | 37 (14.1) | 4 (13.3) | 29 (16.5) | 4 (7.0) |  |
| Second trimester (kg/wk) | 0.69 ± 0.21 | 0.76 ± 0.22 | 0.71 ± 0.20 | 0.63 ± 0.25 | 0.011 |
| Inadequate | 109 (41.4) | 11 (36.7) | 70 (39.8) | 28 (49.1) | 0.083 |
| Adequate | 127 (48.3) | 14 (46.7) | 93 (52.8) | 20 (35.1) |  |
| Excessive | 27 (10.3) | 5 (16.7) | 13 (7.4) | 9 (15.8) |  |
| Third trimester (kg/wk) | 0.70 ± 0.32 | 0.81 ± 0.42 | 0.69 ± 0.31 | 0.69 ± 0.30 | 0.139 |
| Inadequate | 94 (35.7) | 12 (40.0) | 67 (38.1) | 15 (26.3) | 0.007 |
| Adequate | 111 (42.2) | 6 (20.0) | 80 (45.5) | 25 (43.9) |  |
| Excessive | 58 (22.1) | 12 (40.0) | 29 (16.5) | 17 (29.8) |  |

GWG, gestational weight gain; GWGR, gestational weight gain rate.

**Supplementary Table 3.** The concordance rate of growth pattern in MZ and DZ groups.

| Time-point | Group | Concordance^a^ | Discordance^b^ | Rate of Concordance |
| --- | --- | --- | --- | --- |
| 6 mo | MZ | 96 | 22 | 0.814 |
|  | DZ | 94 | 22 | 0.810 |
| 12 mo | MZ | 95 | 15 | 0.864 |
|  | DZ | 88 | 17 | 0.838 |
| 24 mo | MZ | 96 | 7 | 0.932 |
|  | DZ | 81 | 14 | 0.853 |

^a^Concordance: In a twin pair, both infants were normal or overweight. ^b^Discordance: In a twin pair, one infant was normal and his/her sibling was overweight.

**Supplementary Figure 1.** Estimation and 95% CI for GWGR in first trimester with birthweight, BAZ at 6 months, 12 months and 24 months by restricted cubic spline linear regression models among total population.

**
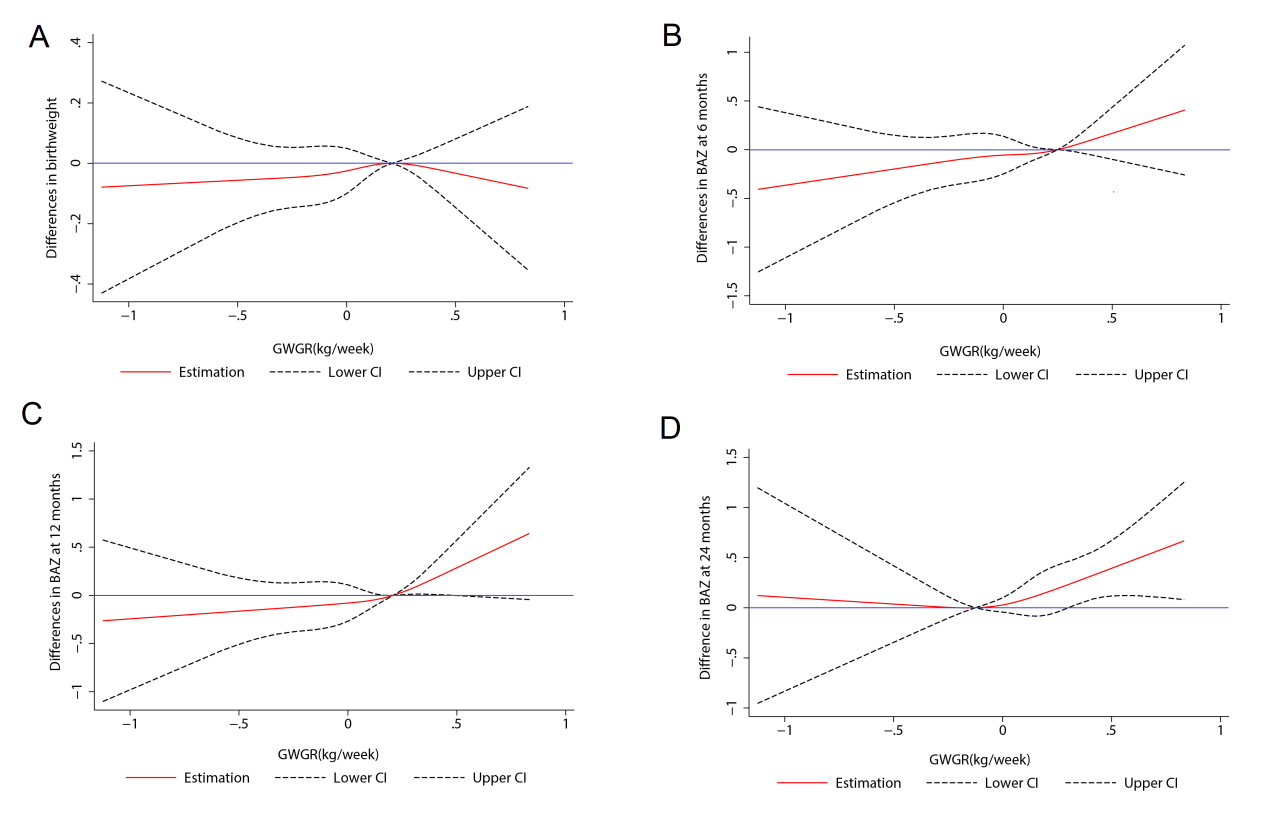
**

BAZ: body mass index z scores; CI: confidence interval; GWGR: gestational weight gain rate.

**Supplementary Figure 2.** Estimation and 95% CI for GWGR in second trimester with birthweight, BAZ at 6 months, 12 months and 24 months by restricted cubic spline linear regression models among total population.

**
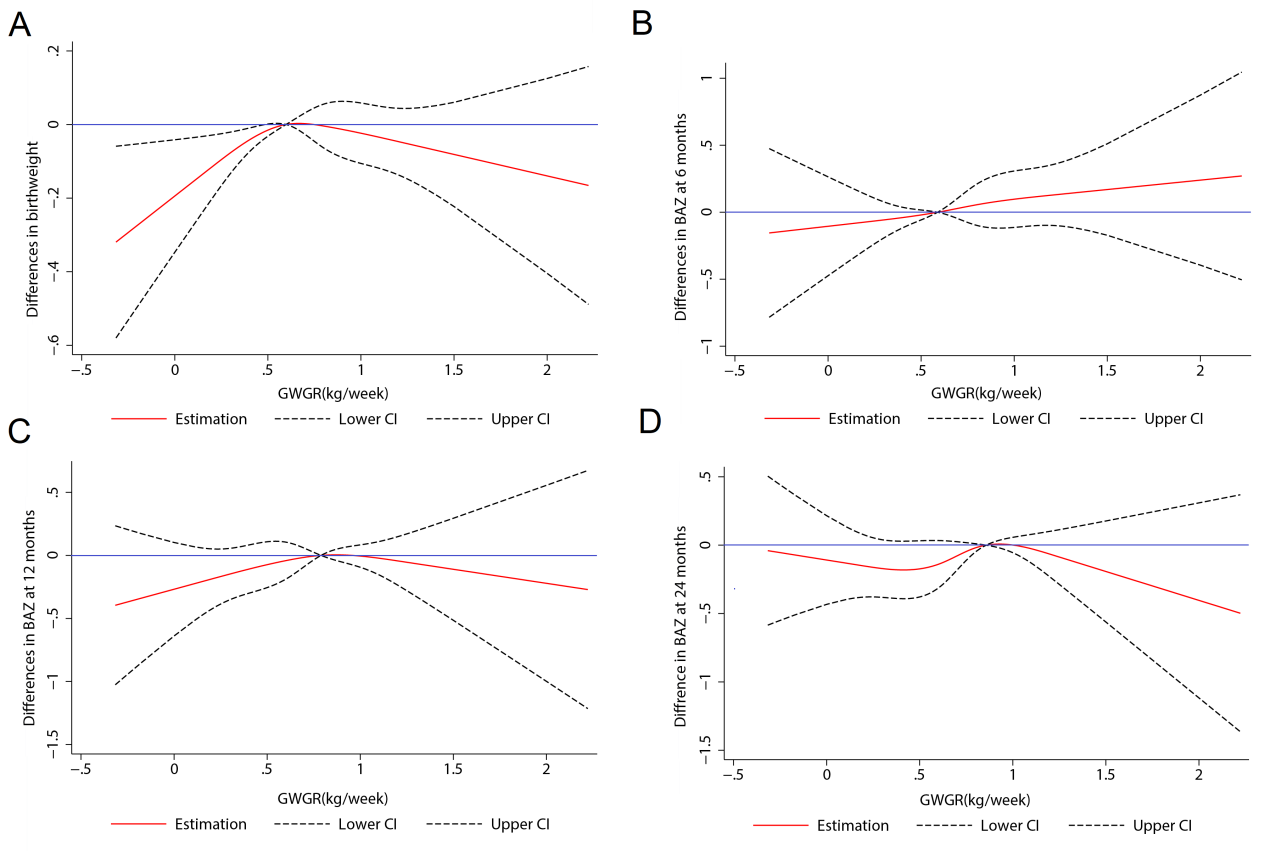
**

BAZ: body mass index z scores; CI: confidence interval; GWGR: gestational weight gain rate.

**Supplementary Figure 3.** Estimation and 95% CI for GWGR in third trimester with birthweight, BAZ at 6 months,12 months and 24 months by restricted cubic spline linear regression models among total population.

**
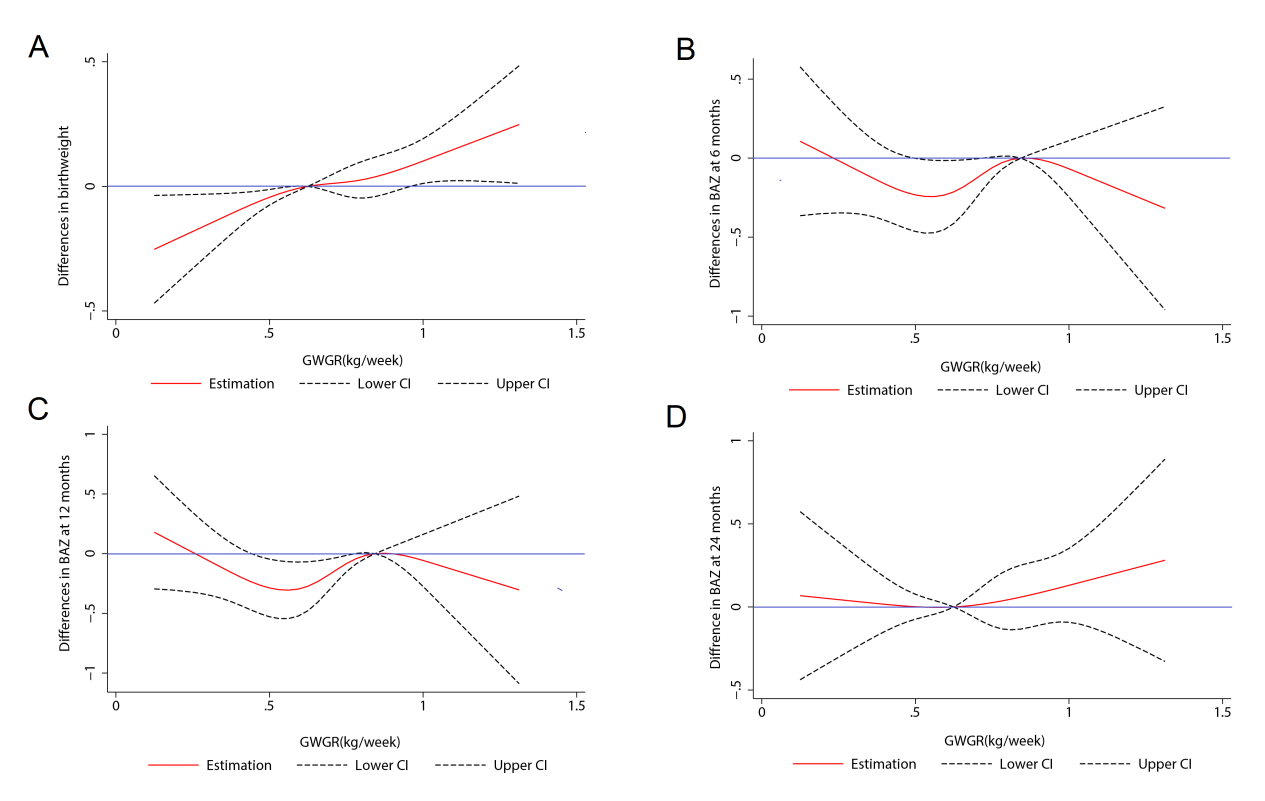
**

BAZ: body mass index z scores; CI: confidence interval; GWGR: gestational weight gain rate.

**Supplementary Figure 4.** Adjusted associations between trimester GWGR and birthweight, age-specific BAZ.


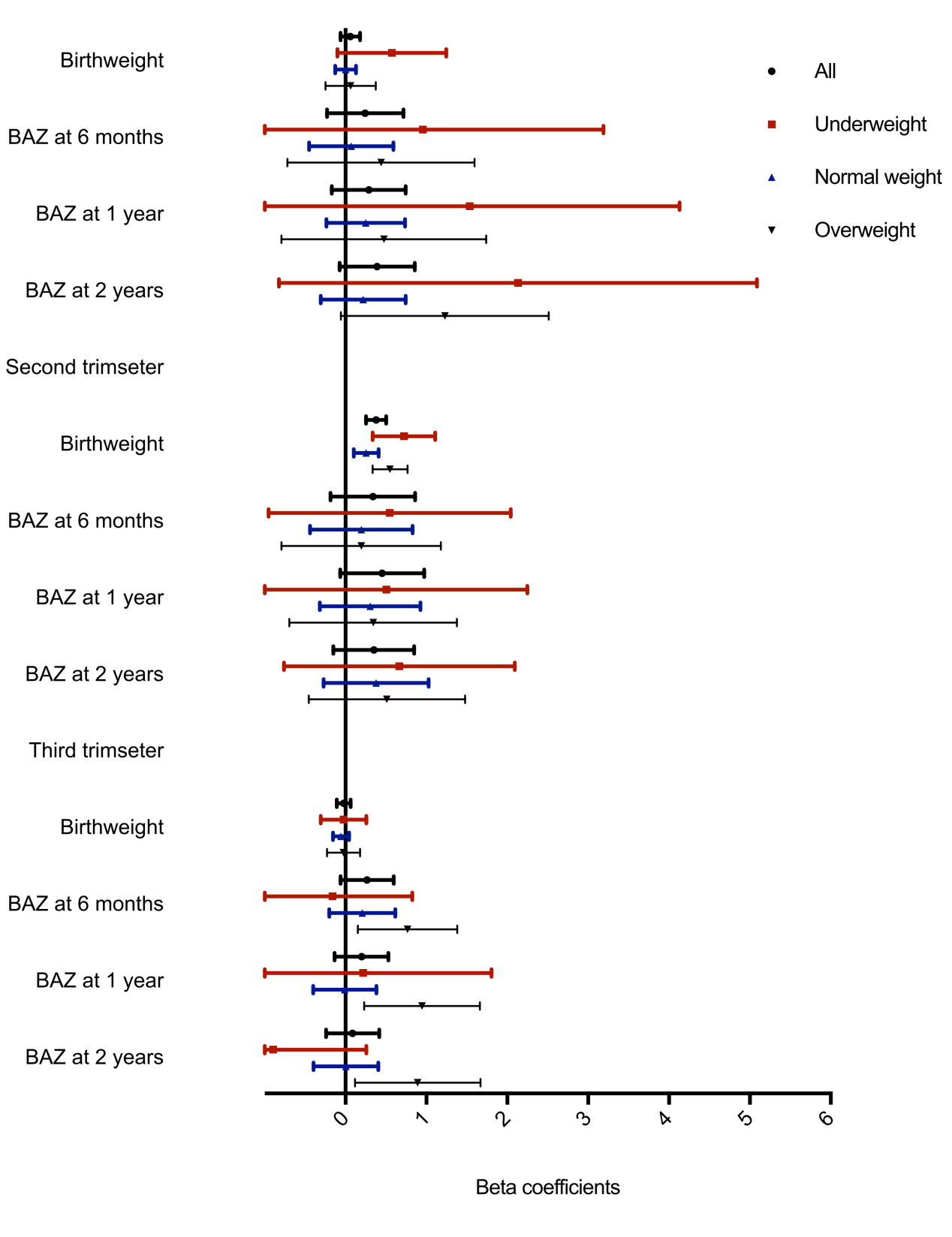


BAZ, body mass index z scores. GWGR: gestational weight gain rate.

Confounding factors including maternal age, prepregnancy BMI, parity, education level, smoking before pregnancy, GDM, gestational age, mode of conception and chorionicity for correlation between trimester GWGR and birthweight.

Confounding factors including maternal age, prepregnancy BMI, parity, education level, smoking before pregnancy, GDM, gestational age, mode of conception, chorionicity and infantile milk feeding patterns within 6 months for correlation between trimester GWGR and age-specific BAZ.
